# Safety of the PCSK9 inhibitor alirocumab: insights from 47 296 patient-years of observation

**DOI:** 10.1093/ehjcvp/pvae025

**Published:** 2024-04-20

**Authors:** Shaun G Goodman, Philippe Gabriel Steg, Michael Szarek, Deepak L Bhatt, Vera A Bittner, Rafael Diaz, Robert A Harrington, J Wouter Jukema, Harvey D White, Andreas M Zeiher, Garen Manvelian, Robert Pordy, Yann Poulouin, Wanda Stipek, Genevieve Garon, Gregory G Schwartz, ODYSSEY OUTCOMES Investigators, Gregory G Schwartz, Ph Gabriel Steg, Deepak L Bhatt, Vera A Bittner, Rafael Diaz, Shaun G Goodman, Robert A Harrington, J Wouter Jukema, Michael Szarek, Harvey D White, Andreas M Zeiher, Pierluigi Tricoci, Matthew T Roe, Kenneth W Mahaffey, Jay M Edelberg, Corinne Hanotin, Guillaume Lecorps, Angèle Moryusef, Robert Pordy, William J Sasiela, Jean-François Tamby, Rafael Diaz, Philip E Aylward, Heinz Drexel, Peter Sinnaeve, Mirza Dilic, Renato D Lopes, Nina N Gotcheva, Shaun G Goodman, Juan-Carlos Prieto, Huo Yong, Patricio López-Jaramillo, Ivan Pećin, Zeljko Reiner, Petr Ostadal, Steen Hvitfeldt Poulsen, Margus Viigimaa, Markku S Nieminen, Nicolas Danchin, Vakhtang Chumburidze, Nikolaus Marx, Evangelos Liberopoulos, Pablo Carlos Montenegro Valdovinos, Hung-Fat Tse, Robert Gabor Kiss, Denis Xavier, Doron Zahger, Marco Valgimigli, Takeshi Kimura, Hyo Soo Kim, Sang-Hyun Kim, Andrejs Erglis, Aleksandras Laucevicius, Sasko Kedev, Khalid Yusoff, Gabriel Arturo Ramos López, Marco Alings, Harvey D White, Sigrun Halvorsen, Roger M Correa Flores, Rody G Sy, Andrzej Budaj, Joao Morais, Maria Dorobantu, Yuri Karpov, Arsen D Ristic, Terrance Chua, Jan Murin, Zlatko Fras, Anthony J Dalby, José Tuñón, H Asita de Silva, Emil Hagström, Ulf Landmesser, Chern-En Chiang, Piyamitr Sritara, Sema Guneri, Alexander Parkhomenko, Kausik K Ray, Patrick M Moriarty, Bernard Chaitman, Sheryl F Kelsey, Anders G Olsson, Jean-Lucien Rouleau, Maarten L Simoons, Karen Alexander, Chiara Meloni, Robert Rosenson, Eric J G Sijbrands, Pierluigi Tricoci, John H Alexander, Luciana Armaganijan, Akshay Bagai, Maria Cecilia Bahit, J Matthew Brennan, Shaun Clifton, Adam D DeVore, Shalonda Deloatch, Sheila Dickey, Keith Dombrowski, Grégory Ducrocq, Zubin Eapen, Patricia Endsley, Arleen Eppinger, Robert W Harrison, Connie Ng Hess, Mark A Hlatky, Joseph Dedrick Jordan, Joshua W Knowles, Bradley J Kolls, David F Kong, Sergio Leonardi, Linda Lillis, Renato D Lopes, David J Maron, Kenneth W Mahaffey, Jill Marcus, Robin Mathews, Rajendra H Mehta, Robert J Mentz, Chetan B Patel, Sabrina Bernardez Pereira, Lynn Perkins, Thomas J Povsic, Etienne Puymirat, Matthew T Roe, William Schuyler Jones, Bimal R Shah, Matthew W Sherwood, Kenya Stringfellow, Darin Sujjavanich, Mustafa Toma, Charlene Trotter, Sean F P van Diepen, Matthew D Wilson, Andrew Tze-Kay Yan, Lilia B Schiavi, Marcelo Garrido, Andrés F Alvarisqueta, Sonia A Sassone, Anselmo P Bordonava, Alberto E Alves De Lima, Jorge M Schmidberg, Ernesto A Duronto, Orlando C Caruso, Leonardo P Novaretto, Miguel Angel Hominal, Oscar R Montaña, Alberto Caccavo, Oscar A Gomez Vilamajo, Alberto J Lorenzatti, Luis R Cartasegna, Gustavo A Paterlini, Ignacio J Mackinnon, Guillermo D Caime, Marcos Amuchastegui, Oscar Salomone, Oscar R Codutti, Horacio O Jure, Julio O E Bono, Adrian D Hrabar, Julio A Vallejos, Rodolfo A Ahuad Guerrero, Federico Novoa, Cristian A Patocchi, Cesar J Zaidman, Maria E Giuliano, Ricardo D Dran, Marisa L Vico, Gabriela S Carnero, Pablo N Guzman, Juan C Medrano Allende, Daniela F Garcia Brasca, Miguel H Bustamante Labarta, Sebastian Nani, Eduardo D S Blumberg, Hugo R Colombo, Alberto Liberman, Victorino Fuentealba, Hector L Luciardi, Gabriel D Waisman, Mario A Berli, Ruben O Garcia Duran, Horacio G Cestari, Hugo A Luquez, Jorge A Giordano, Silvia S Saavedra, Gerardo Zapata, Osvaldo Costamagna, Susana Llois, Jonathon H Waites, Nicholas Collins, Allan Soward, Philip E Aylward, Chris L S Hii, Philip E Aylward, James Shaw, Margaret A Arstall, John Horowitz, Daniel Ninio, James F Rogers, David Colquhoun, Romulo E Oqueli Flores, Philip Roberts-Thomson, Owen Raffel, Sam J Lehman, Constantine Aroney, Steven G M Coverdale, Paul J Garrahy, Gregory Starmer, Mark Sader, Patrick A Carroll, Ronald Dick, Robert Zweiker, Uta Hoppe, Heinz Drexel, Kurt Huber, Rudolf Berger, Georg Delle-Karth, Bernhard Frey, Dirk Faes, Kurt Hermans, Bruno Pirenne, Attilio Leone, Etienne Hoffer, Peter Sinnaeve, Mathias C M Vrolix, Luc De Wolf, Bart Wollaert, Marc Castadot, Karl Dujardin, Christophe Beauloye, Geert Vervoort, Harry Striekwold, Carl Convens, John Roosen, Emanuele Barbato, Marc Claeys, Frank Cools, Ibrahim Terzic, Fahir Barakovic, Zlatko Midzic, Belma Pojskic, Emir Fazlibegovic, Mirza Dilic, Azra Durak-Nalbantic, Mehmed Kulić, Dusko Vulic, Adis Muslibegovic, Boris Goronja, Gilmar Reis, Luciano Sousa, Jose C Nicolau, Flavio E Giorgeto, Ricardo P Silva, Lilia Nigro Maia, Rafael Rech, Paulo R F Rossi, Maria José A G Cerqueira, Norberto Duda, Renato Kalil, Adrian Kormann, José Antonio M Abrantes, Pedro Pimentel Filho, Ana Priscila Soggia, Mayler O N de Santos, Fernando Neuenschwander, Luiz C Bodanese, Yorghos L Michalaros, Freddy G Eliaschewitz, Maria H Vidotti, Paulo E Leaes, Roberto V Botelho, Sergio Kaiser, Euler Roberto F Fernandes Manenti, Dalton B Precoma, Jose C Moura Jorge, Pedro Silva, Jose A Silveira, Wladmir Saporito, Jose A Marin Neto, Gilson S Feitosa, Luiz Eduardo F Ritt, Juliana A de Souza, Fernando Costa, Weimar K S B Souza, Helder J L Reis, Renato D Lopes, Leandro Machado, José Carlos Aidar Ayoub, Georgi V Todorov, Fedya P Nikolov, Elena S Velcheva, Maria L Tzekova, Haralambi O Benov, Stanislav L Petranov, Haralin S Tumbev, Nina S Shehova-Yankova, Dimitar T Markov, Dimitar H Raev, Mihail N Mollov, Kostadin N Kichukov, Katya A Ilieva-Pandeva, Nina N Gotcheva, Raya Ivanova, Maryana Gospodinov, Valentina M Mincheva, Petar V Lazov, Bojidar I Dimov, Manohara Senaratne, James Stone, Jan Kornder, Danielle Dion, Daniel Savard, Yves Pesant, Amritanshu Pandey, Simon Robinson, Gilbert Gosselin, Saul Vizel, Gordon Hoag, Ronald Bourgeois, Anne Morisset, Eric Sabbah, Bruce Sussex, Simon Kouz, Paul MacDonald, Ariel Diaz, Nicolas Michaud, David Fell, Tycho Vuurmans, Christopher Lai, Frank Nigro, Richard Davies, Gustavo Nogareda, Ram Vijayaraghavan, John Ducas, Serge Lepage, Shamir Mehta, James Cha, Robert Dupuis, Peter Fong, Sohrab Lutchmedial, Josep Rodes-Cabau, Hussein Fadlallah, David Cleveland, Thao Huynh, Iqbal Bata, Adnan Hameed, Cristian Pincetti, Sergio Potthoff, Juan C Prieto, Monica Acevedo, Arnoldo Aguirre, Margarita Vejar, Mario Yañez, Guillermo Araneda, Mauricio Fernandez, Luis Perez, Paola Varleta, Fernando Florenzano, Laura Huidobro, Carlos A Raffo, Claudia Olivares, Leonardo Nahuelpan, Humberto Montecinos, Jiyan Chen, Yugang Dong, Weijian Huang, Jianzhong Wang, Shi'An Huang, Zhuhua Yao, Xiang Li, Lan Cui, Wenhua Lin, Yuemin Sun, Jingfeng Wang, Jianping Li, Xuelian Zhang, Hong Zhu, Dandan Chen, Lan Huang, Shaohong Dong, Guohai Su, Biao Xu, Xi Su, Xiaoshu Cheng, Jinxiu Lin, Wenxia Zong, Huanming Li, Yi Feng, Dingli Xu, Xinchun Yang, Yuannan Ke, Xuefeng Lin, Zheng Zhang, Zeqi Zheng, Zhurong Luo, Yundai Chen, Chunhua Ding, Yi Zhong, Yang Zheng, Xiaodong Li, Daoquan Peng, Shuiping Zhao, Ying Li, Xuebo Liu, Meng Wei, Shaowen Liu, Yihua Yu, Baiming Qu, Weihong Jiang, Yujie Zhou, Xingsheng Zhao, Zuyi Yuan, Ying Guo, Xiping Xu, Xubo Shi, Junbo Ge, Guosheng Fu, Feng Bai, Weiyi Fang, Xiling Shou, Xiangjun Yang, Jian'An Wang, Meixiang Xiang, Yingxian Sun, Qinghua Lu, Ruiyan Zhang, Jianhua Zhu, Yizhou Xu, Zhongcai Fan, Tianchang Li, Chun Wu, Nicolas Jaramillo, Gregorio Sanchez Vallejo, Diana C Luna Botia, Rodrigo Botero Lopez, Dora I Molina De Salazar, Alberto J Cadena Bonfanti, Carlos Cotes Aroca, Juan Diego Higuera, Marco Blanquicett, Sandra I Barrera Silva, Henry J Garcia Lozada, Julian A Coronel Arroyo, Jose L Accini Mendoza, Ricardo L Fernandez Ruiz, Alvaro M Quintero Ossa, Fernando G Manzur Jatin, Aristides Sotomayor Herazo, Jeffrey Castellanos Parada, Rafael Suarez Arambula, Miguel A Urina Triana, Angela M Fernandez Trujillo, Maja Strozzi, Siniša Car, Melita Jerić, Martina Lovrić Benčić, Hrvoje Pintarić, Đeiti Prvulović, Jozica Šikić, Viktor Peršić, Dean Mileta, Kresimir Štambuk, Zdravko Babić, Vjekoslav Tomulic, Josip Lukenda, Stanka Mejic-Krstulovic, Boris Starcevic, Jindrich Spinar, David Horak, Zdenek Velicka, Josef Stasek, David Alan, Vilma Machova, Ales Linhart, Vojtech Novotny, Vladimir Kaucak, Richard Rokyta, Robert Naplava, Zdenek Coufal, Vera Adamkova, Ivo Podpera, Jiri Zizka, Zuzana Motovska, Ivana Marusincova, Premysl Svab, Petr Ostadal, Petr Heinc, Jiri Kuchar, Petr Povolny, Jiri Matuska, Steen H Poulsen, Bent Raungaard, Peter Clemmensen, Lia E Bang, Ole May, Morten Bøttcher, Jens D Hove, Lars Frost, Gunnar Gislason, John Larsen, Peter Betton Johansen, Flemming Hald, Peter Johansen, Jørgen Jeppesen, Tonny Nielsen, Kjeld S Kristensen, Piotr Maria Walichiewicz, Jens D Lomholdt, Ib C Klausen, Peter Kaiser Nielsen, Flemming Davidsen, Lars Videbaek, Margus Viigimaa, Mai Soots, Veiko Vahula, Anu Hedman, Üllar Soopõld, Kaja Märtsin, Tiina Jurgenson, Arved Kristjan, Markku S Nieminen, Heikki Huikuri, Pierre Coste, Emile Ferrari, Nicolas Danchin, Olivier Morel, Gilles Montalescot, Jacques Machecourt, Gilles Barone-Rochette, Jacques Mansourati, Yves Cottin, Ph Gabriel Steg, Florence Leclercq, Abdelkader Belhassane, Nicolas Delarche, Franck Boccara, Franck Paganelli, Jérôme Clerc, Francois Schiele, Victor Aboyans, Vincent Probst, Jacques Berland, Thierry Lefèvre, Bernard Citron, Vakhtang Chumburidze, Irakli Khintibidze, Tamaz Shaburishvili, Zurab Pagava, Ramaz Ghlonti, Zaza Lominadze, George Khabeishvili, Rayyan Hemetsberger, Kemala Edward, Ursula Rauch-Kröhnert, Matthias Stratmann, Karl-Friedrich Appel, Ekkehard Schmidt, Heyder Omran, Christoph Stellbrink, Thomas Dorsel, Emmanouil Lianopoulos, Hans Friedrich Vöhringer, Roger Marx, Andreas Zirlik, Detlev Schellenberg, Thomas Heitzer, Ulrich Laufs, Christian Werner, Nikolaus Marx, Stephan Gielen, Sebastian Nuding, Bernhard Winkelmann, Steffen Behrens, Karsten Sydow, Mahir Karakas, Gregor Simonis, Thomas Muenzel, Nikos Werner, Stefan Leggewie, Dirk Böcker, Rüdiger Braun-Dullaeus, Nicole Toursarkissian, Michael Jeserich, Matthias Weißbrodt, Tim Schaeufele, Joachim Weil, Heinz Völler, Johannes Waltenberger, Mohammed Natour, Susanne Schmitt, Dirk Müller-Wieland, Stephan Steiner, Lothar Heidenreich, Elmar Offers, Uwe Gremmler, Holger Killat, Werner Rieker, Sotiris Patsilinakos, Athanasios Kartalis, Athanassios Manolis, Dimitrios Sionis, Geargios Chachalis, Evangelos Liberopoulos, Ioannis Skoumas, Vasilios Athyros, Panagiotis Vardas, Frangkiskos Parthenakis, Dimitrios Alexopoulos, Georgios Hahalis, John Lekakis, Apostolos Hatzitolios, Sergio R Fausto Ovando, Pablo Carlos Montenegro Valdovinos, Juan L Arango Benecke, Edgar R Rodriguez De Leon, Bryan P Y Yan, David C W Siu, Tibor Turi, Bela Merkely, Robert Gabor Kiss, Imre Ungi, Geza Lupkovics, Lajos Nagy, András Katona, István Édes, Gábor Müller, Iván Horvath, Tibor Kapin, Zsolt Szigeti, József Faluközy, Mukund Kumbla, Manjinder Sandhu, Sharath Annam, Naveen Reddy Proddutur, Reddy Regella, Rajendra K Premchand, Ajaykumar Mahajan, Sudhir Pawar, Atul D Abhyanakar, Prafulla Kerkar, Ravishankar A Govinda, Abraham Oomman, Dhurjati Sinha, Sachin N Patil, Dhiman Kahali, Jitendra Sawhney, Abhijeet B Joshi, Sanjeev Chaudhary, Pankaj Harkut, Santanu Guha, Sanjay Porwal, Srimannarayana Jujjuru, Ramesh B Pothineni, Minguel R Monteiro, Aziz Khan, Shamanna S Iyengar, Jasprakash Singh Grewal, Manoj Chopda, Mahesh C Fulwani, Dr Aparna Patange, Patil Sachin, Vijay K Chopra, Naresh K Goyal, Rituparna Shinde, Gajendra V Manakshe, Nitin Patki, Sumeet Sethi, Vengatesh Munusamy, Sunil Karnaand Sunil Thanvi, Srilakshmi Adhyapak, Chandrakant Patil, Ulhas Pandurangi, Rishabh Mathur, Jugal Gupta, Suhas Kalashetti, Ajit Bhagwat, Bagirath Raghuraman, Shiv Kumar Yerra, Prasant Bhansali, Rohidas Borse, Patil Rahul, Srihari Das, Vinay Kumar, Jabir Abdullakutty, Shireesh Saathe, Priya Palimkar, Jabir Abdullkutty, Shireesh Sathe, Shaul Atar, Michael Shechter, Morris Mosseri, Yaron Arbel, Chorin Ehud, Havakuk Ofer, Chaim Lotan, Uri Rosenschein, Amos Katz, Yaakov Henkin, Adi Francis, Marc Klutstein, Eugenia Nikolsky, Robert Zukermann, Yoav Turgeman, Majdi Halabi, Alon Marmor, Ran Kornowski, Michael Jonas, Offer Amir, Yonathan Hasin, Yoseph Rozenman, Shmuel Fuchs, Vered Zvi, Osamah Hussein, Dov Gavish, Zvi Vered, Yoseph Caraco, Mazen Elias, Naveh Tov, Efrat Wolfovitz, Michael Lishner, Nizar Elias, Giancarlo Piovaccari, Annamaria De Pellegrin, Raffaella Garbelotto, Gabriele Guardigli, Valgimigli Marco, Giovanni Licciardello, Carla Auguadro, Filippo Scalise, Claudio Cuccia, Alessandro Salvioni, Giuseppe Musumeci, Michelle Senni, Paolo Calabrò, Salvatore Novo, Pompilio Faggiano, Marco Metra, Nicoletta B De Cesare, Sergio Berti, Claudio Cavallini, Enrico Puccioni, Marcello Galvani, Maurizio Tespili, Piermarco Piatti, Michela Palvarini, Giuseppe De Luca, Roberto Violini, Alessandro De Leo, Zoran Olivari, Pasquale Perrone Filardi, Maurizio Ferratini, Vittorio Racca, Kazuoki Dai, Yuji Shimatani, Haruo Kamiya, Kenji Ando, Yoshihiro Takeda, Yoshihiro Morino, Yoshiki Hata, Kazuo Kimura, Koichi Kishi, Ichiro Michishita, Hiroki Uehara, Toshinori Higashikata, Atsushi Hirayama, Keiji Hirooka, Yasuji Doi, Satoru Sakagami, Shuichi Taguchi, Akihiro Koike, Hiroyuki Fujinaga, Shinji Koba, Ken Kozuma, Tomohiro Kawasaki, Yujiro Ono, Masatoshi Shimizu, Yousuke Katsuda, Atsuyuki Wada, Toshiro Shinke, Takeshi Kimura, Junya Ako, Kenshi Fujii, Toshiyuki Takahashi, Tomohiro Sakamoto, Koichi Nakao, Yutaka Furukawa, Hiroshi Sugino, Ritsu Tamura, Toshiaki Mano, Masaaki Uematsu, Noriaki Utsu, Kashima Ito, Takuya Haraguchi, Katsuhiko Sato, Yasunori Ueda, Akira Nishibe, Kazuteru Fujimoto, Motomaru Masutani, Akira Nishibe, Kazuteru Fujimoto, Jung Han Yoon, Sang-Hyun Kim, Hack-Lyoung Kim, Hun Sik Park, In-Ho Chae, Moo Hyun Kim, Myung Ho Jeong, Seungwoon Rha, Chongjin Kim, Hyo-Soo Kim, Hae Young Kim, Taekjong Hong, Seung-Jea Tahk, Youngkwon Kim, Arija Busmane, Natalija Pontaga, Aldis Strelnieks, Iveta Mintale, Iveta Sime, Zaneta Petrulioniene, Roma Kavaliauskiene, Ruta Jurgaitiene, Gintare Sakalyte, Rimvydas Slapikas, Sigute Norkiene, Nerijus Misonis, Aleksandras Kibarskis, Raimondas Kubilius, Stojko Bojovski, Sasko Kedev, Nensi Lozance, Aleksandar Kjovkaroski, Snezana Doncovska, Tiong Kiam Ong, Sazzli Kasim, Oteh Maskon, Balachandran Kandasamy, Khalid Yusoff, Houng B Liew, Wan Mohd Izani Wan Mohamed, Armando García Castillo, Gabriel Arturo Ramos López, Jorge Carrillo Calvillo, Pedro Fajardo Campos, Juan Carlos Núñez Fragoso, Edmundo Alfredo Bayram Llamas, Marco Antonio Alcocer Gamba, Jaime Carranza Madrigal, Luis Gerardo González Salas, Enrique López Rosas, Belinda González Díaz, Eduardo Salcido Vázquez, Alfredo Nacoud Ackar, Guillermo Antonio Llamas Esperón, Carlos Rodolfo Martínez Sánchez, María Guerrero De Leon, Rodrigo Suarez Otero, Guillermo Fanghänel Salmón, Jesús Antonio Pérez Ríos, José Angel Garza Ruíz, Marco Alings, Robert W Breedveld, Margriet Feenema-Aardema, Alida Borger-Van Der Burg, Pieter A M Hoogslag, Harry Suryapranata, Antonius Oomen, Paulus Van Haelst, Margriet Feenema-Aradema, Jacobijne J Wiersma, Dirk Basart, Ruud M A Van Der Wal, Peter Zwart, Pascalle Monraats, Henricus Van Kesteren, Ioannis Karalis, Johan Jukema, Gerardus J E Verdel, Bart R G Brueren, Roland P T h Troquay, Eric P Viergever, Nadea Y Y Al-Windy, Gerard L Bartels, Jan H Cornel, Walter R M Hermans, Johannes P R Herrman, Robert J Bos, Reginald G E J Groutars, Coenraad C Van Der Zwaan, Refik Kaplan, Raymond Lionarons, Eelko Ronner, Bjorn E Groenemeijer, Patrick N A Bronzwaer, Anho A H Liem, Bernard J W M Rensing, Marcel J J A Bokern, Remco Nijmeijer, Ferry M R J Hersbach, Frank F Willems, Antonius T M Gosselink, Saman Rasoul, John Elliott, Gerard Wilkins, Raewyn Fisher, Douglas Scott, Hamish Hart, Ralph Stewart, Scott Harding, Ian Ternouth, Nicholas Fisher, Samuel Wilson, Denise Aitken, Russell Anscombe, Laura Davidson, Tadeusz Tomala, Ottar Nygård, Jon Arne Sparby, Kjell Andersen, Lars Gullestad, Jarle Jortveit, Peter S Munk, Erlend gyllensten Singsaas, Sigrun Halvorsen, Ulf Hurtig, Roger M Correa Flores, Jorge R Calderon Ticona, Julio R Durand Velasquez, Sandra A Negron Miguel, Enrique S Sanabria Perez, Jesus M Carrion Chambilla, Carlos A Chavez Ayala, Reynaldo P Castillo Leon, Rolando J Vargas Gonzales, Jose D Hernandez Zuniga, Luis A Camacho Cosavalente, Jorge E Bravo Mannucci, Javier Heredia Landeo, Nassip C Llerena Navarro, Yudy M Roldan Concha, Víctor E Rodriguez Chavez, Henry A Anchante Hernandez, Carlos A Zea Nunez, Walter Mogrovejo Ramos, Arthur Ferrolino, Rosa Allyn G Sy, Louie Tirador, Rody G Sy, Generoso Matiga, Raul Martin Coching, Alisa Bernan, Gregorio Rogelio, Dante D Morales, Edgar Tan, Dennis Jose Sulit, Adrian Wlodarczak, Krystyna Jaworska, Grzegorz Skonieczny, Lidia Pawlowicz, Pawel Wojewoda, Benita Busz-Papiez, Janusz Bednarski, Aleksander Goch, Pawel Staneta, Elzbieta Dulak, Andrzej Budaj, Krzysztof Saminski, Wlodzimierz Krasowski, Wanda Sudnik, Aleksander Zurakowski, Marcin Skorski, Roman Lysek, Beata Miklaszewicz, Jacek Kubica, Jan Andrzej Lipko, Edyta Kostarska-Srokosz, Marek Piepiorka, Anna Drzewiecka, Ryszard Sciborski, Arkadiusz Stasiewski, Tomasz Blicharski, Leszek Bystryk, Michal Szpajer, Marek Korol, Tomasz Czerski, Ewa Mirek-Bryniarska, Jacek Gniot, Andrzej Lubinski, Jerzy Gorny, Edward Franek, Grzegorz Raczak, Hanna Szwed, Pedro Monteiro, Jose Mesquita Bastos, Helder H Pereira, Dinis Martins, Joao Morais, Filipe Seixo, Carlos Mendonça, Ana Botelho, Francisca Caetano, Bogdan Minescu, Octavian Istratoaie, Dan N Tesloianu, Maria Dorobantu, Gabriel Cristian, Silviu Dumitrescu, Cristian G C Podoleanu, Mircea C A Constantinescu, Cristina M Bengus, Constantin Militaru, Doina Rosu, Irinel R Parepa, Adrian V Matei, Tom M Alexandru, Mihaela Malis, Ioan Coman, Rodica Stanescu-Cioranu, Doina Dimulescu, Yury Shvarts, Olga Orlikova, Zhanna Kobalava, Olga L Barbarash, Valentin Markov, Nadezhda Lyamina, Alexander Gordienko, Konstantin Zrazhevsky, Alexander Y Vishnevsky, Victor Gurevich, Raisa Stryuk, Nikita V Lomakin, Igor Bokarev, Tatiana Khlevchuk, Sergey Shalaev, Larisa Khaisheva, Petr Chizhov, Inna Viktorova, Natalya Osokina, Vladimir Shchekotov, Evgenia Akatova, Galina Chumakova, Igor Libov, Mikhail I Voevoda, Tatyana V Tretyakova, Evgeny Baranov, Sergey Shustov, Sergey Yakushin, Ivan Gordeev, Niiaz Khasanov, Olga Reshetko, Tatiana Sotnikova, Olga Molchanova, Konstantin Nikolaev, Liudmila Gapon, Elena Baranova, Zaur Shogenov, Elena Kosmachova, Yuriy Karpov, Yuri Karpov, Anton Povzun, Liudmila Egorova, Vadim V Tyrenko, Igor G Ivanov, Masterov Ilya, Sergey Kanorsky, Dragan Simic, Nikola Ivanovic, Goran Davidovic, Nebojsa Tasic, Milika R Asanin, Stevo Stojic, Svetlana R Apostolovic, Stevan Ilic, Biljana Putnikovic Tosic, Aleksandar Stankovic, Aleksandra Arandjelovic, Slavica Radovanovic, Branislava Todic, Arsen D Ristic, Jovan Balinovac, Dragan V Dincic, Petar Seferovic, Ana Karadzic, Slobodan Dodic, Sinisa Dimkovic, Tamara Jakimov, Terrance Chua, Kian-Keong Poh, Hean Yee Ong, Justin Tang I-Shing, Karol Micko, Jan Nociar, Daniel Pella, Peter Fulop, Marian Hranai, Juraj Palka, Juraj Mazur, Ivan Majercák, Andrej Dzupina, František Fazekas, Jozef Gonsorcik, Viliam Bugan, Jan Murin, Juraj Selecky, Gabriel Kamensky, Jaroslava Strbova, Rudolf Smik, Andrej Dukat, Peter Olexa, Ivan Žuran, Janez Poklukar, Nataša Černič Šuligoj, Matija Cevc, Zlatko Fras, Henry P Cyster, Naresh Ranjith, Clive Corbett, Junaid Bayat, Ellen Makoali Makotoko, Hendrik du Toit Theron, Ilse E Kapp, Matthys M de V Basson, Hanlie Lottering, Dina Van Aswegen, Louis J Van Zyl, Peter J Sebastian, Thayabran Pillay, Jan A Saaiman, Patrick J Commerford, Soraya Cassimjee, Garda Riaz, Iftikhar O Ebrahim, Mahomed Sarvan, Joseph H Mynhardt, Anthony J Dalby, Helmuth Reuter, Rajendran Moodley, Manuel Vida, Angel R Cequier Fillat, Vicente Bodí Peris, Francisco Fuentes Jimenez, Francisco Marín, Jose M Cruz Fernández, Rafael Jesus Hidalgo Urbano, Blas Gil-Extremera, Pablo Toledo, Fernando Worner Diz, David Garcia-Dorado, Andres Iñiguez, José Tuñón Fernández, Jose R Gonzalez-Juanatey, Javier Fernandez Portales, Fernando Civeira Murillo, Laia Matas Pericas, Jose Luis Zamorano, Manuel De Mora Martin, Jordi Bruguera Cortada, Joaquin J Alonso Martin, Jose Maria Serrano Antolin, José R De Berrazueta Fernández, José Antonio Vázquez de Prada, Jose Francisco Díaz Fernández, José Alberto García Lledó, Juan Cosín Sales, Javier Botas Rodriguez, Gabriel Gusi Tragant, Amparo Benedicto, Carlos Gonzalez-Juanatey, Mercedes Camprubí Potau, Ignacio Plaza Perez, César Morís De La Tassa, Pablo Loma-Osorio Rincon, Javier Balaguer Recena, Juan M Escudier, Antonio Coca Payeras, Norberto Alonso Orcajo, Pedro Valdivielso, Godwin Constantine, Ruvaiz Haniffa, Nirmali Tissera, Stanley Amarasekera, Chandrike Ponnamperuma, Nimali Fernando, Kaputella Fernando, Jayanthimala Jayawardena, Santharaj Wijeyasingam, Gotabhaya Ranasinghe, Ruvan Ekanayaka, Sepalika Mendis, Vajira Senaratne, Gnanamoorthy Mayurathan, Ajantha Rajapaksha, Thilak Sirisena, Jagath I Herath, Naomali Amarasena, Stefan Berglund, Gundars Rasmanis, Emil Hagström, Ola Vedin, Nils Witt, Georgios Mourtzinis, Peter Nicol, Ole Hansen, Stefano Romeo, Steen Agergaard Jensen, Ingemar Torstensson, Ulf Ahremark, Torbjörn Sundelin, Tiziano Moccetti, Christian Müller, Francois Mach, Ronald Binde, Ulf Landmesser, Oliver Gämperli, Chern-En Chiang, Wei-Chuan Tsai, Kwo-Chang Ueng, Wen-Ter Lai, Ming-En Liu, Juey-Jen Hwang, Wei-Hsian Yin, I-Chang Hsieh, Ming-Jer Hsieh, Wei Hsiang Lin, Jen-Yuan Kuo, Tsuei-Yuan Huang, Chih-Yuan Fang, Pinij Kaewsuwanna, Wasant Soonfuang, Woravut Jintapakorn, Apichard Sukonthasarn, Piyamitr Sritara, Nattawut Wongpraparut, Krisada Sastravaha, Nakarin Sansanayudh, Wirash Kehasukcharoen, Dilok Piyayotai, Paiboon Chotnoparatpat, Ahmet Camsari, Hakan Kultursay, Sema Guneri, Bulent Mutlu, Murat Ersanli, Mustafa Demirtas, Cevat Kirma, Ertan Ural, Lale Koldas, Oleksandr Karpenko, Alexander Prokhorov, Ihor Vakaluyk, Halyna Myshanych, Dmytro Reshotko, Valeriy Batushkin, Leonid Rudenko, Ihor Kovalskyi, Mykola Kushnir, Vira Tseluyko, Yuriy Mostovoy, Mykola Stanislavchuk, Yulian Kyiak, Yuriy Karpenko, Yaroslav Malynovsky, Andriy Klantsa, Oles Kutniy, Ekaterina Amosova, Viktor Tashchuk, Oleh Leshchuk, Alexander Parkhomenko, Mykola Rishko, Mykola Kopytsya, Andriy Yagensky, Mykola Vatutin, Andriy Bagriy, Olga M Barna, Olexiy Ushakov, Georgiy Dzyak, Borys Goloborodko, Anatolii Rudenko, Volodymyr Zheleznyy, Jasper Trevelyan, Azfar Zaman, Kaeng Lee, Andrew Moriarty, Rajesh K Aggarwal, Piers Clifford, Yuk-Ki Wong, Syed M R Iqbal, Eduardas Subkovas, Denise Braganza, David Sarkar, Robert Storey, Huw Griffiths, Sam Mcclure, Rangasamy Muthusamy, Simon Smith, John Kurian, Terry Levy, Craig Barr, Honer Kadr, Robert Gerber, Audrius Simaitis, Handrean Soran, Anthony Mathur, Adrian Brodison, Mohammad Ayaz, Muhammad Cheema, Richard Oliver, Simon Thackray, Telal Mudawi, Gohar Rahman, Ayyaz Sultan, Timothy Reynolds, David Sharman, david Sprigings, Rob Butler, Peter Wilkinson, Gregory Y H Lip, Julian Halcox, Sean Gallagher, Nicholas Ossei-Gerning, Gil Vardi, Duccio Baldari, David Brabham, Charles Treasure, Charles Dahl, Bruce Palmer, Alan Wiseman, Abul Khan, Sanjeev Puri, Ann Elizabeth Mohart, Carlos Ince, Enrique Flores, Scott Wright, Shi-Chi Cheng, Michael Rosenberg, William Rogers, Edward Kosinski, Les Forgosh, Jonathan Waltman, Misal Khan, Mohammad Shoukfeh, Georges Dagher, Patrick Cambier, Ira Lieber, Priya Kumar, Cara East, Perry Krichmar, Mian Hasan, Lindsey White, Thomas Knickelbine, Thomas Haldis, Eve Gillespie, Thomas Amidon, David Suh, Imran Arif, mouhamad Abdallah, Faiq Akhter, Eric Carlson, Michael D'Urso, Fadi El-Ahdab, William Nelson, Katie Moriarty, Barry Harris, Steven Cohen, Luther Carter, Daniel Doty, Kenneth Sabatino, Tariq Haddad, Amir Malik, Sunder Rao, Angel Mulkay, Ion Jovin, Kim Klancke, Vinay Malhotra, Sai K Devarapalli, Michael Koren, Harish Chandna, George Dodds, Tauqir Goraya, James Bengston, Matthew Janik, Joseph Moran, Andrew Sumner, John Kobayashi, William Davis, Shahram Yazdani, John Pasquini, Maitreya Thakkar, Amarnath Vedere, Wayne Leimbach, James Rider, Sarah fenton, Narendra Singh, Anil V Shah, Patrick M Moriarty, Denise Janosik, Carl Pepine, Brett Berman, Joseph Gelormini, Christopher Daniels, Kerensky Richard, Friederike Keating, Nicholas I Kondo, Sanjay Shetty, Howard Levite, Winfried Waider, Theodore Takata, Mazen Abu-Fadel, Vipul Shah, Rahul Aggarwal, Mark Izzo, Anil Kumar, Brack Hattler, Rose Do, Chad Link, Anna Bortnick, George Kinzfogl, Arnold Ghitis, John Larry, Edward Teufel, Peter Kuhlman, Brent Mclaurin, Wenwu Zhang, Stephen Thew, Jalal Abbas, Matthew White, Othman Islam, Sumeet Subherwal, Nandkishore Ranadive, Babak Vakili, Christian Gring, David Henderson, Timothy Schuchard, Naim Farhat, Geoffrey Kline, Sharan Mahal, Jack Whitaker, Shawn Speirs, Rolf Andersen, Nizar Daboul, Phillip Horwitz, Firas Zahr, George Ponce, Zubair Jafar, Joseph Mcgarvey, Vipul Panchal, Stephen Voyce, Thomas Blok, William Sheldon, Masoud M Azizad, Carsten Schmalfuss, Mark Picone, Robert Pederson, William Herzog, Keith Friedman, Jason Lindsey, Rosemary Nowins, Eichenlaub Timothy, Parilak Leonard, Norman Lepor, Mahfouz El Shahawy, Howard Weintraub, Anand Irimpen, Alvaro Alonso, Wade May, Daniels Christopher, Thomas Galski, Alan Chu, Freny Mody, Ebrahimi Ramin, Zachary Hodes, Joseph Rossi, Gregory Rose, James Fairlamb, Charles Lambert, Ajit Raisinghani, Antonio Abbate, George Vetrovec, Marilyn King, Charles Carey, Jaime Gerber, Liwa Younis, Hyeun (Tom) Park, Mladen Vidovich, Thomas Knutson, Dennis Friedman, Fred Chaleff, Arthur Loussararian, Phillip Rozeman, Carey Kimmelstiel, Jeffrey Kuvin, Kevin Silver, Malcolm Foster, Glen Tonnessen, Andrey Espinoza, Mohamadali Amlani, Andreas Wali, Christopher Malozzi, Geert T Jong, Clara Massey, Keattiyoat Wattanakit, Philip J O'Donnell, Dinesh Singal, Naseem Jaffrani, Sridhar Banuru, Daniel Fisher, Mark Xenakis, Neal Perlmutter, Ravi Bhagwat, James Strader, Ronald Blonder, Ayim Akyea-Djamson, Ajay Labroo, Kwan Lee, H John Marais, Edmund Claxton, Robert Weiss, Rohr Kathryn, Martin Berk, Peter Rossi, Parag Joshi, Amit Khera, Ajit S Khaira, Greg Kumkumian, Steven Lupovitch, Joshua Purow, Stephen Welka, David Hoffman, Stuart Fischer, Eugene Soroka, Donald Eagerton, Samir Pancholy, Michael Ray, Norman Erenrich, Michael Farrar, Stewart Pollock, William J French, Steve Diamantis, Douglas Guy, Lawrence Gimple, Mark Neustel, Steven Schwartz, Edward Pereira, Seals Albert, Douglas Spriggs, Janet Strain, Suneet Mittal, Anthony Vo, Majed Chane, Jason Hall, Nampalli Vijay, Kapildeo Lotun, F Martin Lester, Ahed Nahhas, Theodore Pope, Paul Nager, Rakesh Vohra, Mukesh Sharma, Riyaz Bashir, Hinan Ahmed, Michael Berlowitz, Robert Fishberg, Robert Barrucco, Eric Yang, Michael Radin, Daniel Sporn, Dwight Stapleton, Steven Eisenberg, Joel Landzberg, Martin Mcgough, Samir Turk, Michael Schwartz, P Sandy Sundram, Diwakar Jain, Mark Zainea, Carlos Bayron, Ronald Karlsberg, Suhail Dohad, Henry Lui, William Keen, Donald Westerhausen, Sandeep Khurana, Himanshu Agarwal, Jessica Birchem, William Penny, Mark Chang, Sherrill Murphy, John Henry, Branislav Schifferdecker, John M Gilbert, Gopal Chalavarya, Charles Eaton, John F Schmedtje, Stuart Christenson, Imran Dotani, Douglas Denham, Alexander Macdonell, Paul Gibson, Aref Rahman, Tammam Al Joundi, Nizar Assi, Gary Conrad, Purushotham Kotha, Michael Love, Gregory Giesler, Howard Rubenstein, Dawood Gamil, Laura Akright, Justine Krawczyk, Joanne Cobler, Terry Wells, James Welker, Robert Foster, Richard Gilmore, Jay Anderson, Douglas Jacoby, Bill Harris, Geraldine Gardner, Ramprasad Dandillaya, Kishor Vora, John Kostis, John Hunter, David Laxson, Eric Ball, Jay Anderson, Terry Wells, Kishor Vora, Eric Ball, James Welker, Renato D Lopes, Flavia Egydio, Anelise Kawakami, Janaina Oliveira, Shaun G Goodman, Julianna Wozniak, Alexander Matthews, Caroline Ratky, Janine Valiris, Lisa Berdan, Anita Hepditch, Kirby Quintero, Matthew T Roe, Tyrus Rorick, Melissa Westbrook, Rafael Diaz, Andrea Pascual, Carla Rovito, Nicolas Danchin, Madeleine Bezault, Elodie Drouet, Tabassome Simon, Harvey D White, Caroline Alsweiler, Peter Sinnaeve, Anne Luyten, Philip E Aylward, Julie Butters, Liddy Griffith, Michelle Shaw, Emil Hagström, Lena Grunberg, Michael Szarek, Shahidul Islam, Marie-France Brégeault, Nathalie Bougon, Douglas Faustino, Sylvie Fontecave, Judith Murphy, Jean-Francois Tamby, Melanie Verrier, Veronique Agnetti, Dorthe Andersen, Emmy Badreddine, Mhamed Bekkouche, Cecile Bouancheau, Imane Brigui, Maddy Brocklehurst, Joseph Cianciarulo, Dawn Devaul, Szilvia Domokos, Cecile Gache, Caroline Gobillot, Severine Guillou, Jan Healy, Megan Heath, Gayatri Jaiwal, Carine Javierre, Julien Labeirie, Myriam Monier, Ulises Morales, Asmaa Mrabti, Bicky Mthombeni, Betim Okan, Lucile Smith, Jennifer Sheller, Sebastien Sopena, Valerie Pellan, Fadela Benbernou, Nafissa Bengrait, Maud Lamoureux, Katarina Kralova, Michel Scemama, Raphael Bejuit, Anthony Coulange, Christelle Berthou, Jérôme Repincay, Christelle Lorenzato, Alexis Etienne, Valerie Gouet, Guillaume Lecorps, Virginie Loizeau, Mickael Normand, Anne Ourliac, Christelle Rondel, Antony Adamo, Pascale Beltran, Pauline Barraud, Helene Dubois-Gache, Benjamin Halle, Lamia Metwally, Maxime Mourgues, Marc Sotty, Marion Vincendet, Raluca Cotruta, Zhu Chengyue, Dominique Fournie-Lloret, Christine Morrello, Aurelie Perthuis, Patrick Picault, Isabelle Zobouyan, Helen M Colhoun, Michael A Dempsey, Mark A McClanahan

**Affiliations:** Canadian VIGOUR Centre, University of Alberta, Edmonton, Alberta, Canada; St. Michael's Hospital, University of Toronto, Toronto, Ontario, Canada; Université Paris-Cité, Institut Universitaire de France, Assistance Publique-Hôpitaux de Paris, Hôpital Bichat, FACT (French Alliance for Cardiovascular Trials), and INSERM U1148, F-75018 Paris, France; CPC Clinical Research and Division of Cardiology, University of Colorado School of Medicine, Aurora, 80045 CO, USA; State University of New York, Downstate Health Sciences University, Brooklyn, NY 11203, USA; Mount Sinai Heart, Icahn School of Medicine at Mount Sinai, New York, NY 10029, USA; Division of Cardiovascular Disease, University of Alabama at Birmingham, Birmingham, AL 35233, USA; Estudios Cardiológicos Latinoamérica, Instituto Cardiovascular de Rosario, S2000 Rosario, Argentina; Cornell University, New York, NY 10065, USA; Department of Cardiology, Leiden University Medical Center, 2333 ZA Leiden, The Netherlands; Netherlands Heart Institute, 3511 EP Utrecht, The Netherlands; Green Lane Cardiovascular Research Unit, Te Whatu Ora—Health New Zealand, Te Toka Tumai, and University of Auckland, Auckland 1030, New Zealand; Department of Medicine III, Goethe University, 60596 Frankfurt am Main, Germany; Regeneron Pharmaceuticals Inc., Tarrytown, NY 10591, USA; Regeneron Pharmaceuticals Inc., Tarrytown, NY 10591, USA; IT&M Stats, Neuilly-sur-Seine 92200, France; Regeneron Pharmaceuticals Inc., Tarrytown, NY 10591, USA; Sanofi, Toronto, ON M2R 3T4, Canada; University of Colorado School of Medicine, Aurora 80045, CO, USA

**Keywords:** Safety, Alirocumab, PCSK9, Cholesterol

## Abstract

The ODYSSEY OUTCOMES trial, comprising over 47 000 patient-years of placebo-controlled observation, demonstrated important reductions in the risk of recurrent ischaemic cardiovascular events with the monoclonal antibody to proprotein convertase subtilisin/kexin type 9 alirocumab, as well as lower all-cause death. These benefits were observed in the context of substantial and persistent lowering of low-density lipoprotein cholesterol with alirocumab compared with that achieved with placebo. The safety profile of alirocumab was indistinguishable from matching placebo except for a ∼1.7% absolute increase in local injection site reactions. Further, the safety of alirocumab compared with placebo was evident in vulnerable groups identified before randomization, such as the elderly and those with diabetes mellitus, previous ischaemic stroke, or chronic kidney disease. The frequency of adverse events and laboratory-based abnormalities was generally similar to that in placebo-treated patients. Thus, alirocumab appears to be a safe and effective lipid-modifying treatment over a duration of at least 5 years.

## Graphical Abstract

**Graphical Abstract:**
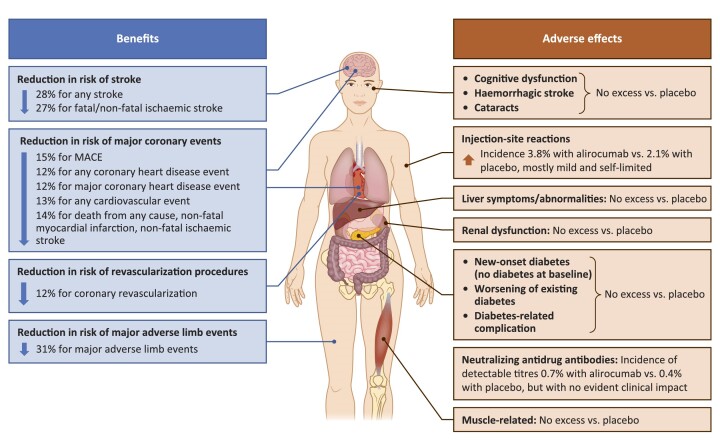
Overview of the clinical efficacy and safety of alirocumab as observed in the ODYSSEY OUTCOMES clinical trial. MACE, major adverse cardiovascular event.

## Introduction

Alirocumab is a monoclonal antibody to proprotein convertase subtilisin/kexin type 9 (PCSK9)^1^ approved by most international regulatory agencies for the treatment of hypercholesterolaemia in individuals who require additional lowering of low-density lipoprotein cholesterol (LDL-C). The short- (e.g. 8 weeks) to medium-term (e.g. 52−104 weeks) lipid-lowering efficacy and safety of alirocumab compared with placebo or ezetimibe were established in 14 randomized, double-blind trials involving 5234 subjects.^2–20^ The clinical efficacy of alirocumab over a median follow-up of 2.8 years and a maximum follow-up of 5 years was firmly established in the ODYSSEY OUTCOMES trial, which involved 18 924 patients with recent acute coronary syndrome (ACS) and elevated atherogenic lipoprotein levels despite high-intensity or maximum-tolerated statin treatment.^21^

Concerns regarding intensive lowering of cholesterol have, however, been raised, given that cholesterol is the main component of cell membranes and intracellular structures, serves as a precursor in the biosynthesis of some vitamins, steroids, and sex hormones, and plays a key role in hepatic bile production.^22^ Further, some studies have found an association between low cholesterol levels and an increased risk for dementia, intracranial haemorrhage, and death. The Further cardiovascular Outcomes Research with PCSK9 Inhibition in 27 564 subjects with Elevated Risk (FOURIER) trial demonstrated that the PCSK9 monoclonal antibody evolocumab could significantly lower LDL-C and the risk of cardiovascular events in patients with stable atherosclerotic cardiovascular disease followed for a median of 2.2 years without differences in major safety events or cognitive function testing when compared with placebo.^23–25^ At the conclusion of randomized treatment, 6635 (24%) participants from both treatment arms continued in an open-label extension study and received evolocumab for a total observation time up to 8 years.^26^ Extended treatment with evolocumab in the group initially randomized to evolocumab was safe, well-tolerated, and associated with fewer cardiovascular events and cardiovascular deaths compared with delayed treatment with evolocumab in those initially randomized to placebo who survived the parent study.^26,27^ To review and extend the findings in the ODYSSEY OUTCOMES trial, this report provides safety data from 47 296 patient-years of observation after randomized assignment to treatment with alirocumab or placebo.

To date, reports from ODYSSEY OUTCOMES have focused on the clinical efficacy of alirocumab (reduction of cardiovascular events and association with numerically fewer deaths)^21,28–30^ across prespecified subgroups.^21,31–35^ Although, in aggregate, no safety concerns with alirocumab have emerged, specific categories of patients may be particularly vulnerable to adverse events and safety outcomes, such as the elderly and those with diabetes mellitus, previous ischaemic stroke, or chronic kidney disease (*Graphical abstract*). This report reviews the overall safety findings in the trial and then examines the safety and tolerability of alirocumab in the specific subgroups indicated above, as well as the effect of alirocumab on key laboratory tests.

## Summary of overall safety findings from the ODYSSEY OUTCOMES trial

### Adverse events and laboratory abnormalities

**Figure 1:**
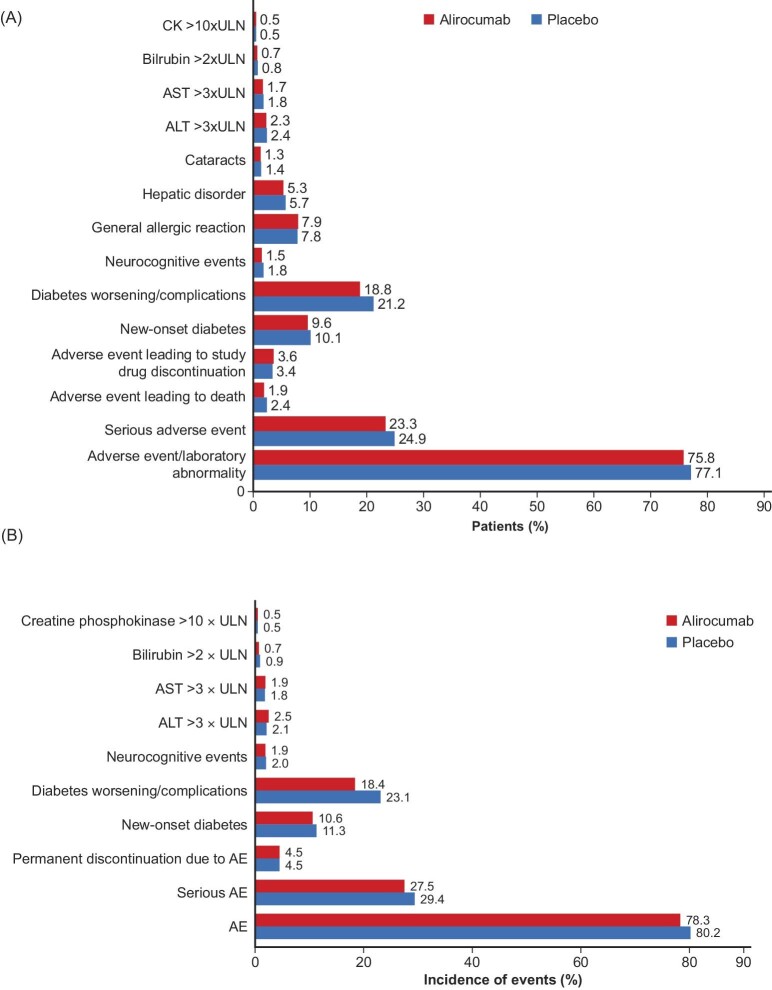
Frequency of laboratory abnormalities and adverse events in the (*A*) overall patient population and (*B*) subgroup of 8228 patients (43.5%) eligible for 3−5 years of treatment (modified with permission) in the ODYSSEY OUTCOMES trial.^21,36^ ALN, alanine transaminase; AST, aspartate aminotransferase; CK, creatine kinase; ULN, upper limit of normal.

**Figure 2:**
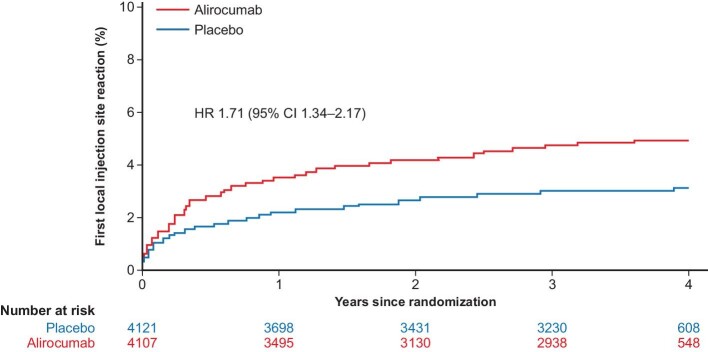
Kaplan–Meier cumulative incidence for time to first local injection site reaction in the subgroup of 8228 patients (43.5%) eligible for 3−5 years of treatment over ∼4 years.^36^ CI, confidence interval; HR, hazard ratio. (Reproduced with permission).

In the ODYSSEY OUTCOMES trial over a median (quartile [Q]1, Q3) follow-up of 2.8 (2.3−3.4) years, alirocumab was essentially indistinguishable from placebo with respect to the frequency of adverse events, serious adverse events, adverse events leading to death, adverse events leading to study drug discontinuation, general allergic reaction, hepatic disorder, and cataracts (*Figure 1**A*).^21^ The only exception was patient-reported local injection site reactions, which occurred more frequently in the alirocumab group (3.8% vs. 2.1%; *P* < 0.001). However, these injection site reactions (e.g. itching, erythema, or swelling) were usually mild, self-limited, and led to study drug discontinuation in only 26 (of 9462) alirocumab-treated patients at a median of 8.3 months after randomization vs. 3 patients in the placebo group.^21^ Further, among 8242 patients (43.5%) eligible for 3–5 years of follow-up (i.e. randomized ≥3 years before the common study end date),^29^ 8228 received one or more doses of study medication, comprising 24 610 patient-years of observation, with a median follow-up of 3.3 years.^36^ The Kaplan–Meier cumulative incidence for time to first local injection site reaction in this subgroup was <5% over ∼4 years, with most occurring within the first 6 months (*Figure 2*).^36^ Treatment-emergent adverse events occurred in 78.3% of alirocumab- and 80.2% of placebo-treated patients in this subgroup, including 27.5% and 29.4% of serious adverse events, respectively; treatment-emergent adverse events leading to death occurred in 2.7% and 3.3% in the alirocumab and placebo groups, respectively (*Figure 1**B*).

Recognizing that the ODYSSEY OUTCOMES trial employed a single-blind placebo run-in period (2−16 weeks) for eligible patients to be instructed in the technique of self-injection of study drug using a 1 mL prefilled pen,^37^ which could lead to a more adherent population, premature discontinuation of the assigned alirocumab or placebo for reasons other than death occurred in 1343 patients (14.2%) in the alirocumab group and 1496 patients (15.8%) in the placebo group over the median follow-up of 2.8 years. In the subgroup eligible for 3−5 years of follow-up, rates of permanent treatment discontinuation due to adverse events were similar in both treatment groups (*Figure 1**B*).

The incidence of laboratory abnormalities was similar in the alirocumab and placebo groups (*Figure 1**A*). Alanine (ALT) and aspartate (AST) aminotransferases, total bilirubin, and creatine kinase (CK) were monitored serially in the setting of 89% of patients receiving high-intensity atorvastatin (80 or 40 mg in 27%) or rosuvastatin (40 or 20 mg in 62%) as background lipid-lowering treatment. There were infrequent and similar incidences of elevations of these laboratory tests [ALT and AST >3 times upper limit of normal (ULN), bilirubin >2 times ULN, CK >10 times ULN] during follow-up in the alirocumab and placebo groups.^21^ Similarly, among a prespecified subgroup of 8228 patients eligible for 3−5 years of follow-up, elevations of ALT >3 (2.5% vs. 2.1%), AST >3 (1.9% vs. 1.8%), bilirubin >2 (0.7% vs. 0.9%), and CK >10 (0.5% vs. 0.5%) times ULN were similar in the alirocumab and placebo groups, respectively (*Figure 1**B*). The low incidence of transaminase and CK elevations is also notable in this population where ∼83–87% of patients remained on high-intensity statin treatment at 1 and 3 years post-randomization.^21^

## Alirocumab in vulnerable populations in the ODYSSEY OUTCOMES trial

Alirocumab was not associated with an excess of laboratory abnormalities or adverse events compared with placebo in any patient subgroup including the elderly and those with diabetes or chronic kidney disease.

### Older patients

The mean age at entry into the ODYSSEY OUTCOMES trial (58 years) was somewhat younger than in most cardiovascular outcome trials because of the selection of patients with ACS based upon elevated levels of atherogenic lipoproteins, reflecting a lifetime risk factor and thus a younger age at presentation with disease. Nonetheless, ODYSSEY OUTCOMES enrolled 5084 (26.9%) patients ≥65 years of age, of whom 1007 (5.3%) were ≥75 years of age and 42 (0.2%) were ≥85 years of age. Prespecified subgroup analyses comparing the efficacy and safety of alirocumab vs. placebo were undertaken, stratified according to younger (40−64 years) and older (≥65 years) age.^21,34^ Older patients were more often female and more likely to have a history of hypertension, diabetes, myocardial infarction, percutaneous coronary intervention (PCI) or coronary artery bypass grafting (CABG), stroke, peripheral artery disease, and heart failure (HF).^34^ Older patients were also more likely to have presented with non-ST-segment elevation myocardial infarction (vs. ST-segment elevation myocardial infarction or unstable angina) and less likely to have undergone coronary revascularization (PCI or CABG) for their index ACS event. Adherence to assigned study treatment decreased over time in both age categories (e.g. ∼88% in patients <65 years of age vs. ∼86% in patients ≥65 years of age at 2 years) but was similar in the alirocumab and placebo groups. The relative benefit of alirocumab over placebo on the primary (and key secondary) outcome was consistent across the entire age range, with estimated absolute benefit increasing with advancing age due to higher absolute risk. Consistent with the safety findings we observed in those ≥65 years of age,^21^ although adverse events were observed more frequently in patients aged ≥75 years compared with the younger cohort, there were no differences between alirocumab and placebo in older or younger patients (*Table 1*). Similarly, serious adverse events or adverse events that led to discontinuation of the trial regimen were more frequent in older patients, but without differences between the randomized treatment arms. Patient/site-reported neurocognitive disorders were infrequent (<2% overall), and although numerically more common among patients ≥75 years of age, the incidence did not differ between the alirocumab and placebo groups in this age stratum [age ≥65 years: 2.4% vs. 4.7%; hazard ratio (HR): 0.52, 95% confidence interval (CI): 0.26−1.03; age <75 years: 1.5% vs. 1.6%; HR: 0.91, 95% CI: 0.72−1.16)]. Thus, in the ODYSSEY OUTCOMES trial, adding alirocumab to maximum-tolerated high-intensity statins significantly improved outcomes in patients after an ACS irrespective of age, without any age-related safety issues.^34^

**Table 1:** Adverse events by randomized treatment and age group (≥75 vs. <75 years old).

	Randomized treatment		
Adverse event	Alirocumab, *n*/*N* (%)	Placebo, *n*/*N* (%)	Relative risk (95% CI) (alirocumab vs. placebo)
Any adverse events			
≥75 years old	391/492 (79.5)	439/513 (85.6)	0.93 (0.88−0.98)
<75 years old	6774/8959 (75.6)	6843/8930 (76.6)	0.99 (0.97−1.00)
Serious adverse events			
≥75 years old	163/492 (33.1)	194/513 (37.8)	0.88 (0.74−1.04)
<75 years old	2039/8959 (22.8)	2156/8930 (24.1)	0.94 (0.89−0.99)
Adverse event that led to discontinuation of the trial regimen			
≥75 years old	22/492 (4.5)	36/513 (7.0)	0.64 (0.38−1.07)
<75 years old	321/8959 (3.6)	288/8930 (3.2)	1.11 (0.95−1.30)
Neurocognitive disorder			
≥75 years old	12/492 (2.4)	24/513 (4.7)	0.52 (0.26−1.03)
<75 years old	131/8959 (1.5)	143/8930 (1.6)	0.91 (0.72−1.16)
New-onset diabetes among patients without diabetes at baseline			
≥75 years old	26/330 (7.9)	37/346 (10.7)	0.74 (0.46−1.19)
<75 years old	622/6433 (9.7)	639/6350 (10.1)	0.96 (0.87−1.07)
Haemorrhagic stroke—adjudicated			
≥75 years old	0/492 (0.0)	2/513 (0.4)	−
<75 years old	9/8959 (0.1)	14/8930 (0.2)	0.64 (0.28−1.48)
Alanine transaminase >3 ULN			
≥75 years old	11/486 (2.3)	15/507 (3.0)	0.77 (0.35−1.65)
<75 years old	201/8883 (2.3)	213/8834 (2.4)	0.94 (0.78−1.14)
Aspartate aminotransferase >3 ULN			
≥75 years old	9/486 (1.9)	10/507 (2.0)	0.94 (0.38−2.29)
<75 years old	151/8881 (1.7)	156/8831 (1.8)	0.96 (0.77−1.20)

CI, confidence interval; ULN, upper limit of normal.

### Diabetes mellitus

In the ODYSSEY OUTCOMES trial at baseline, 5444 (28.8%) patients had a history of diabetes, 8246 (43.6%) prediabetes, and 5234 (27.7%) normoglycaemia.^31^ While patients with diabetes had the highest incidence of the primary outcome over a median of 2.8 years (16.4% vs. 9.2% prediabetes vs. 8.5% normoglycaemia), the relative benefit of alirocumab was consistent across each glycaemic category and the absolute benefit was greatest in those with previous diabetes (i.e. number needed to treat for 3 years, ∼44 vs. 83 vs. 83, respectively).

A blinded independent expert committee was prospectively established to review and adjudicate potential cases of incident diabetes among patients without diabetes at baseline. This was an important objective, given previous observations that statins increase the risk of incident diabetes,^38,39^ and findings in Mendelian randomization analyses that genes encoding variants in PCSK9 associated with lower LDL-C levels are also associated with greater incident diabetes.^40^ In fact, alirocumab did not increase the risk of new-onset diabetes (*Figure 1**A*) among patients without diabetes at baseline (including those with prediabetes). Likewise, among the patients eligible for ≥3 years of follow-up, new-onset diabetes occurred with similar frequency among the alirocumab and placebo groups: 10.6% vs. 11.3% with prediabetes and 3.8% vs. 3.0% with normoglycaemia at baseline (*Figure 1**B*). Despite achieving a median LDL-C of 0.80 mmol/L at 4 months post-randomization, alirocumab had no effect on plasma glucose concentrations or haemoglobin A_1c_. In addition, no increase in diabetes worsening or diabetes complications was observed with alirocumab compared with placebo (*Figure 1**A*) among patients with diabetes at baseline.^31^ Finally, in 2281 patients with diabetes at baseline who were eligible for ≥3 years of follow-up, 18.4% of alirocumab- and 23.1% of placebo-treated patients experienced diabetes worsening or complications, including diabetes-related serious adverse events (but no deaths) in 3.3% of alirocumab- and 3.0% of placebo-treated patients (*Figure 1**B*).^36^

The abovementioned LDL-C and non-high-density lipoprotein cholesterol lowering and safety findings in the subgroup of patients in ODYSSEY OUTCOMES with diabetes mellitus are consistent with those from the ODYSSEY DM-INSULIN^41^ and ODYSSEY DM-DYSLIPIDEMIA^42^ trials comparing alirocumab with placebo both in patients on maximum-tolerated statin and in patients with diabetes on insulin (both type 1 and type 2) and type 2 diabetes, respectively.

Because low levels of lipoprotein(a) have been associated with an increased risk of incident diabetes, and because PCSK9 inhibitors lower levels of lipoprotein(a), the effect of alirocumab vs. placebo on incident diabetes as a function of baseline lipoprotein(a) was evaluated in an additional exploratory analysis of ODYSSEY OUTCOMES.^43^ While there was no overall effect of alirocumab on incident diabetes, patients with low baseline levels of lipoprotein(a) had a lower incidence with alirocumab than with placebo and patients with high baseline lipoprotein(a) levels (∼1.29 mmol/L or greater) tended to have an increased risk of incident diabetes. Nonetheless, the cardiovascular benefits of alirocumab in patients with high lipoprotein(a) in ODYSSEY OUTCOMES^44,45^ appear to outweigh any possible small increase in the risk of incident diabetes in that subset.^43^

### Stroke

**Figure 3:**
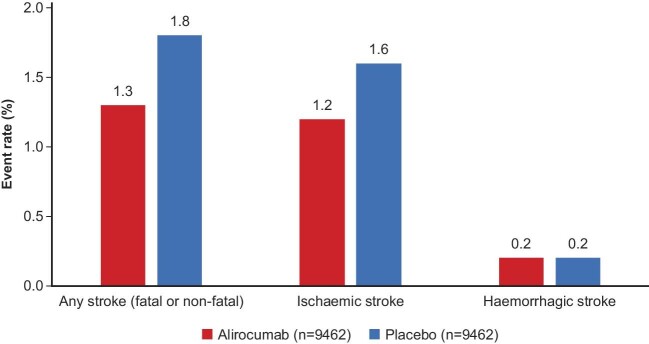
Risk of any fatal or non-fatal stroke, ischaemic stroke, and haemorrhagic stroke in the ODYSSEY OUTCOMES trial.^21,48^

While lowering of atherogenic lipoproteins with statins reduces the risk of ischaemic stroke,^46^ concerns have been raised about the association of spontaneously very low LDL-C levels and the higher risk for haemorrhagic stroke, as well as a potential increase in intracranial haemorrhage in patients receiving intensive statin therapy after ischaemic stroke.^39,47^ In the overall population in the ODYSSEY OUTCOMES trial, with blinded adjudication of all stroke events, alirocumab significantly reduced the risk of any stroke and ischaemic stroke without any increase in haemorrhagic stroke (*Figure 3*).^21,48^ The benefit of alirocumab for reducing the risk of stroke was similar among 944 patients (5.0%) with a history of cerebrovascular disease and among those without a history of cerebrovascular disease (*P*_interaction_ = 0.37).^48^ Furthermore, there was no relation between lower achieved LDL-C (at 4 months post-randomization) and the incidence of haemorrhagic stroke in the alirocumab group.^21,48^ An important caveat is that a history of haemorrhagic stroke was an exclusion criterion in ODYSSEY OUTCOMES and the safety of alirocumab in patients with previous haemorrhagic stroke therefore remains undetermined.

### Chronic kidney disease

In the ODYSSEY OUTCOMES trial, the aggregate baseline estimated glomerular filtration rate (eGFR) was 83 ± 18 mL/min/1.73 m^2^, including 2122 patients (11.2%) with an eGFR <60 mL/min/1.73 m^2^.^35^ While an eGFR <30 mL/min/1.73 m^2^ was a screening exclusion criterion, 69 patients (0.4%) had an eGFR <30 mL/min/1.73 m^2^ at randomization. The annualized incidence rates for the primary outcome and death increased progressively as eGFR decreased, with patients receiving alirocumab having fewer events than those on placebo across all values of eGFR, with larger relative risk reductions in those with eGFR >60 mL/min/1.73 m^2^. Alirocumab had no effect on eGFR over the duration of the trial. The percentages of patients having a decrease in eGFR from baseline of ≥30% (1.8% alirocumab vs. 2.1% placebo; *P* = 0.09), 40% (0.8% vs. 0.9%; *P* = 0.48), or 50% (0.3% vs. 0.4%; *P* = 0.62) were similar in both treatment groups. Further, this subgroup analysis found no excess of any adverse event (other than local injection site reactions) with alirocumab compared with placebo in any category of eGFR.^35^ Therefore, both the efficacy and safety of alirocumab appeared consistent across the eGFR categories enrolled in ODYSSEY OUTCOMES. This finding is consistent with the fact that alirocumab is an immunoglobulin G (IgG) monoclonal antibody; renal elimination is relatively unimportant for IgG, as its large size prevents efficient filtration through the glomerulus. Thus, IgG elimination occurs primarily via intracellular catabolism following receptor-mediated endocytosis; based on non-renal elimination of alirocumab, renal impairment would not be expected to significantly affect the pharmacokinetic/pharmacodynamic profile of alirocumab.

Nevertheless, an important caveat is that the number of patients with advanced chronic kidney disease in ODYSSEY OUTCOMES was too low to allow for meaningful conclusions for patients with an eGFR <30 mL/min/1.73 m², and patients on dialysis were excluded from the trial.

### Neurocognitive events

In 2012, the US Food and Drug Administration (FDA) issued a warning regarding potential adverse effects of statins on neurocognition (e.g. memory loss, confusion) based on the Adverse Events Reporting System and a review of the medical literature. However, a subsequent Mendelian randomization study showed that low LDL-C levels due to 3-hydroxy-3-methylglutaryl-CoA reductase (and PCSK9) genetic variants had no causal effects on the increased risk of Alzheimer's disease, vascular dementia, any dementia, or Parkinson's disease.^49^ Further, evidence (including frequency of adverse cognitive events reported or measurements using standard neuropsychological cognitive test scores) from placebo-controlled randomized clinical trials of statins failed to support any association between cognitive impairment and statin therapy in cognitively normal or impaired subjects.^50^ In the context of this uncertainty, in 2014, the FDA directed pharmaceutical companies conducting clinical trials of PCSK9 inhibitors to carefully monitor cognitive adverse effects. Two meta-analyses of Phase 2 and 3 trials concluded that PCSK9 inhibitors were not associated with an increased risk of severe adverse events, musculoskeletal effects, or stroke,^51^ and potentially reduced all-cause death,^52^ but suggested an increased incidence of adverse neurocognitive effects.^51,52^ Subsequent pooled analyses of 10−14 trials reported no safety concerns with alirocumab treatment over an 8- to 104-week follow-up, even with very low levels of achieved LDL-C,^15,17,20^ including incidence of neurocognitive adverse events.^18^ However, the median and maximum exposure times in these trials were relatively brief. Therefore, a longer-term safety assessment, such as that recently reported,^36^ was deemed desirable.^53^

In the ODYSSEY OUTCOMES trial, with median and maximum observation times of 2.8 and 5.0 years, neurocognitive disorders were reported in 1.5% of alirocumab- and 1.8% of placebo-treated patients (*Figure 1**A*).^21^ In the subgroup of 8228 patients eligible for ≥3 years of follow-up, neurocognitive disorders were reported in 80 (1.9%) patients in the alirocumab group and 83 (2.0%) patients in the placebo group (*Figure 1**B*). While serial neurocognitive testing was not employed in ODYSSEY OUTCOMES, a dedicated neurocognitive study (double-blind, placebo-controlled) of alirocumab in 2176 patients with heterozygous familial hypercholesterolaemia or non-familial hypercholesterolaemia at high/very high cardiovascular risk demonstrated no effect of alirocumab on neurocognitive function assessed using the Cambridge Neuropsychological Test Automated Battery (CANTAB) over 96 weeks of treatment.^54^ These findings are consistent with those from the prospective cognitive function substudy of the FOURIER trial^23^ with evolocumab and the Evaluating PCSK9 Binding Antibody Influence on Cognitive Health in High Cardiovascular Risk Subjects (EBBINGHAUS) study.^25^

### Antidrug antibodies

In the ODYSSEY OUTCOMES trial, 5.5% of patients treated with alirocumab compared with 1.6% of patients treated with placebo had antidrug antibodies detected after initiating treatment, with most of these being transient responses. Persistent antidrug antibody responses (defined by the presence of positive responses detected after the start of study drug administration in two or more consecutive post-baseline serum samples and separated by a ≥16-week period) were observed in 0.7% of patients treated with alirocumab and 0.4% of patients treated with placebo. Neutralizing antibody responses were observed in 0.5% of patients treated with alirocumab and in <0.1% of patients treated with placebo.^21^ Similarly, among patients eligible for ≥3 years of follow-up, antidrug antibodies were observed more frequently in the alirocumab vs. placebo group (0.9% vs. 0.5%); neutralizing antibodies on two or more occasions were observed in only one patient in each group. However, the clinical importance of these findings, particularly in the context of a fully human monoclonal antibody therapy, remains unclear. An analysis of 10 trials involving 4747 patients concluded ‘antidrug antibodies developed in few patients who were treated with alirocumab, and even those patients had substantial and durable evidence of LDL-cholesterol lowering’.^55^ Further, the development—albeit infrequent—of antidrug and neutralizing antibodies in 32 and 6 placebo-treated patients raises uncertainty about the specificity of the anti-alirocumab test itself. Most importantly, there were no discernible safety concerns associated with detection of antidrug antibodies.

## Efficacy and safety of alirocumab according to the achieved level of LDL-C in the ODYSSEY OUTCOMES trial

The optimal LDL-C concentration achieved with lipid-lowering therapies for reducing cardiovascular events with acceptable safety remains uncertain. For example, in a *post hoc* analysis of the Justification for the Use of Statins in Primary Prevention: An Intervention Trial Evaluating Rosuvastatin (JUPITER) trial, patients achieving LDL-C <0.78 mmol/L experienced significant increases in diabetes, haematuria, hepatobiliary disorders, and insomnia.^56^ In contrast, a prespecified analysis of the Improved Reduction of Outcomes: Vytorin Efficacy International Trial (IMPROVE-IT) found that post-ACS patients achieving an LDL-C <0.78 mmol/L at 1 month after randomization (to either simvastatin or simvastatin plus ezetimibe) had a similar safety profile (and numerically the lowest rate of cardiovascular events) over a 6-year period compared with patients achieving higher LDL-C concentrations.^22^ A prespecified regression analysis of the FOURIER trial with the PCSK9 inhibitor evolocumab showed a monotonic relationship between achieved (at 4 weeks) LDL-C and major cardiovascular outcomes down to an LDL-C concentration of <0.2 mmol/L, without association of achieved LDL-C with any safety outcome.^24^ Inference from this regression analysis regarding clinical efficacy as a function of achieved LDL-C should be considered with the caveats that patients in both the placebo and evolocumab groups were included, patients who achieved the lowest LDL-C levels were likely to have started with low baseline LCL-C levels, and that concurrent levels of lipoprotein(a) and study medication adherence were not considered.

An analysis of ODYSSEY OUTCOMES attempted to overcome these limitations by categorizing alirocumab-assigned patients according to three strata of LDL-C achieved at month 4 (<0.65, 0.65–1.29, or >1.29 mmol/L).^57^ Each of these categories was matched in a 1:1 ratio to patients from the placebo group with similar baseline characteristics [including LDL-C and lipoprotein(a)] and study medication adherence, using a propensity score. Treatment HR and the absolute reduction in the risk of major adverse cardiovascular events (MACE) with alirocumab were examined in each category. In the placebo group, there was a gradient in the risk of MACE, with the greatest incidence among those matched to patients with achieved LDL-C >1.29 mmol/L with alirocumab and the lowest incidence among those matched to patients with achieved LDL-C <0.65 mmol/L with alirocumab. Treatment HR and absolute risk reduction were similar for the achieved LDL-C categories of 0.65−1.29 mmol/L and <0.65 mmol/L. For those with achieved LDL-C >1.29 mmol/L with alirocumab, treatment HR was higher and absolute risk reduction lower than for the other categories. The conclusion of this analysis was that an achieved LDL-C of 0.65−1.29 mmol/L may be a reasonable goal after ACS.

Regarding safety according to achieved levels of LDL-C, a pooled analysis of 14 Phase 2 and 3 trials in the ODYSSEY programme, with follow-up as long as 104 weeks, found similar rates of adverse events in alirocumab-treated patients achieving two consecutive LDL-C values <0.65 and <0.39 mmol/L compared with those who did not achieve LDL-C <0.65 mmol/L, including neurological and neurocognitive events.^20^ However, in a propensity score analysis, the rate of cataracts was 0.8% higher in patients achieving an LDL-C level <0.65 mmol/L. No difference in cataract incidence was observed between the pooled alirocumab and control (placebo or ezetimibe) groups.^20^ The incidence of cataracts was similar in the alirocumab and placebo groups in the ODYSSEY OUTCOMES trial (1.3% vs. 1.4%).^58^ Further, in patients treated with alirocumab with two or more LDL-C values of <0.65 mmol/L, the incidence of cataracts was 1.6% vs. 1.4% in propensity score-matched patients from the placebo group.^58^

The ODYSSEY OUTCOMES trial was designed with a treat-to-target approach, with blinded adjustment of the alirocumab dose to maximize the number of patients who achieved LDL-C values of 0.65–1.29 mmol/L and minimize prolonged exposure to levels <0.39 mmol/L.^21,37^ With this caveat, no safety concerns were associated with the relatively limited period of LDL-C <0.39 mmol/L (an average of 6.8 months spent below this level before blinded substitution of placebo at a median 8.3 months from randomization in 730 patients).^57^ This included similar rates of neurocognitive events and haemorrhagic stroke in alirocumab-treated patients achieving these very low LDL-C levels compared with the aggregate placebo group or propensity score-matched patients in the placebo group. The 525 of 6769 (7.8%) patients in the alirocumab group without diabetes at baseline who achieved consecutive LDL-C levels <0.39 mmol/L were at a greater risk of new-onset diabetes than those in the aggregate placebo group (15.1% vs. 10.1%; HR: 1.46, 95% CI: 1.16−1.85; *P* = 0.001). However, this difference in the risk of new-onset diabetes was attenuated and no longer statistically significant compared with the propensity score-matched placebo subgroup without diabetes at baseline (15.1% vs. 13.0%; HR: 1.10, 95% CI: 0.85−1.43; *P* = 0.46).^57^

The overall safety profile of alirocumab in the ODYSSEY OUTCOMES trial appears excellent: the only side effect that occurred more frequently than in the placebo group was mild injection site reactions. However, despite the present analysis representing more than 47 000 patient-years of follow-up, the longest follow-up was 5 years and the mean age of the participating patients at the time of randomization was 59 years. Therefore, it is not possible to exclude the remote possibility that more serious safety signals could emerge over longer periods of treatment and in older more fragile populations. Thus, as with any newer class of drugs, continued pharmacovigilance efforts are warranted. It is reassuring that, since alirocumab has been on the market for approximately 8 years, no serious adverse event has emerged and has been reported in a pharmacovigilance report to various health authorities (e.g. the European Medicines Agency (EMA)) that regularly monitor drug safety.

## Conclusions

The ODYSSEY OUTCOMES trial comprised over 47 000 patient-years of placebo-controlled observation, including observation in 8228 patients eligible for at least 3, and up to 5, years of follow-up and received at least one dose of study medication. The trial demonstrated important reductions in the risk of recurrent ischaemic cardiovascular events with alirocumab, as well as fewer deaths compared with placebo. These benefits were observed in the context of substantial and persistent lowering of LDL-C compared with placebo. The safety profile of alirocumab was indistinguishable from matching placebo except for a ∼1.7% absolute increase in local injection site reactions ∼3 years. No safety concerns with alirocumab emerged in the patients eligible for 3−5 years of follow-up. Further, the safety of alirocumab compared with placebo was evident in vulnerable groups identified before randomization, such as the elderly and those with diabetes mellitus, previous ischaemic stroke, or chronic kidney disease. The frequency of adverse events and laboratory-based abnormalities was generally similar when compared with placebo. Thus, alirocumab appears to be both a safe and effective lipid-modifying treatment over a duration of at least 5 years.

## Supplementary Material

pvae025_Supplemental_File

## Notes

**Conflict of interest:** S.G.G. reports research grant support (e.g. steering committee or data and safety monitoring committee) and/or speaker/consulting honoraria (e.g. advisory boards) from Amgen, AstraZeneca, Bayer, Boehringer Ingelheim, Bristol Myers Squibb, CSL Behring, Daiichi-Sankyo/American Regent, Eli Lilly, Esperion, Ferring Pharmaceuticals, GlaxoSmithKline, HLS Therapeutics, JAMP Pharma, Janssen/Johnson & Johnson, Merck, Novartis, Novo Nordisk A/C, Pendopharm, Pfizer, Regeneron, Sanofi, Servier, Valeo Pharma; and salary support/honoraria from the Heart and Stroke Foundation of Ontario/University of Toronto (Polo) Chair, Canadian Heart Research Centre and MD Primer, Canadian VIGOUR Centre, Cleveland Clinic Coordinating Centre for Clinical Research, Duke Clinical Research Institute, New York University Clinical Coordinating Centre, and PERFUSE Research Institute. P.G.S. reports grants, personal fees, and non-financial support from Sanofi; grants and personal fees from Amarin, Servier, and Bayer; personal fees from Amgen, AstraZeneca, BMS, Boehringer Ingelheim, Idorsia, Pfizer, and Novartis. In addition, P.G.S. has a patent use of alirocumab to reduce risk after ACS (royalties to Sanofi) pending. M.S. reports serving as a consultant or on advisory boards (or both) for CiVi, Resverlogix, Baxter, Esperion, Sanofi, and Regeneron Pharmaceuticals, Inc. D.L.B. discloses the following relationships: advisory board: AngioWave, Bayer, Boehringer Ingelheim, Cardax, CellProthera, Cereno Scientific, Elsevier Practice Update Cardiology, High Enroll, Janssen, Level Ex, McKinsey, Medscape Cardiology, Merck, MyoKardia, NirvaMed, Novo Nordisk, PhaseBio, PLx Pharma, Regado Biosciences, Stasys; board of directors: AngioWave (stock options), Boston VA Research Institute, Bristol Myers Squibb (stock), DRS.LINQ (stock options), High Enroll (stock), Society of Cardiovascular Patient Care, TobeSoft; chair: Inaugural Chair, American Heart Association Quality Oversight Committee; consultant: Broadview Ventures; Data Monitoring Committees: Acesion Pharma, Assistance Publique-Hôpitaux de Paris, Baim Institute for Clinical Research (formerly Harvard Clinical Research Institute, for the PORTICO trial, funded by St. Jude Medical, now Abbott), Boston Scientific (chair, PEITHO trial), Cleveland Clinic (including for the ExCEED trial, funded by Edwards), Contego Medical (chair, PERFORMANCE 2), Duke Clinical Research Institute, Mayo Clinic, Mount Sinai School of Medicine (for the ENVISAGE trial, funded by Daiichi-Sankyo; for the ABILITY-DM trial, funded by Concept Medical), Novartis, Population Health Research Institute; Rutgers University [for the National Institutes of Health (NIH)-funded MINT Trial]; honoraria: American College of Cardiology (Senior Associate Editor, Clinical Trials and News, ACC.org; chair, ACC Accreditation Oversight Committee), Arnold & Porter law firm (work related to Sanofi/Bristol Myers Squibb clopidogrel litigation), Baim Institute for Clinical Research (formerly Harvard Clinical Research Institute; RE-DUAL PCI clinical trial steering committee funded by Boehringer Ingelheim; AEGIS-II executive committee funded by CSL Behring), Belvoir Publications (Editor in Chief, Harvard Heart Letter), Canadian Medical and Surgical Knowledge Translation Research Group (clinical trial steering committees), Cowen and Company, Duke Clinical Research Institute (clinical trial steering committees, including for the PRONOUNCE trial, funded by Ferring Pharmaceuticals), HMP Global (Editor in Chief, *Journal of Invasive Cardiology*), *Journal of the American College of Cardiology* (Guest Editor; Associate Editor), K2P (co-chair, interdisciplinary curriculum), Level Ex, Medtelligence/ReachMD (CME steering committees), MJH Life Sciences, Oakstone CME (course director, Comprehensive Review of Interventional Cardiology), Piper Sandler, Population Health Research Institute (for the COMPASS operations committee, publications committee, steering committee, and USA national co-leader, funded by Bayer), Slack Publications (Chief Medical Editor, Cardiology Today's Intervention), Society of Cardiovascular Patient Care (secretary/treasurer), WebMD (CME steering committees), Wiley (steering committee); other: Clinical Cardiology (deputy editor), NCDR-ACTION Registry Steering Committee (chair), VA CART Research and Publications Committee (chair); patent: sotagliflozin (named on a patent for sotagliflozin assigned to Brigham and Women's Hospital that assigned to Lexicon; neither I nor Brigham and Women's Hospital receive any income from this patent); research funding: Abbott, Acesion Pharma, Afimmune, Aker Biomarine, Amarin, Amgen, AstraZeneca, Bayer, Beren, Boehringer Ingelheim, Boston Scientific, Bristol Myers Squibb, Cardax, CellProthera, Cereno Scientific, Chiesi, CinCor, CSL Behring, Eisai, Ethicon, Faraday Pharmaceuticals, Ferring Pharmaceuticals, Forest Laboratories, Fractyl, Garmin, HLS Therapeutics, Idorsia, Ironwood, Ischemix, Janssen, Javelin, Lexicon, Lilly, Medtronic, Merck, Moderna, MyoKardia, NirvaMed, Novartis, Novo Nordisk, Owkin, Pfizer, PhaseBio, PLx Pharma, Recardio, Regeneron, Reid Hoffman Foundation, Roche, Sanofi, Stasys, Synaptic, The Medicines Company, Youngene, 89Bio; royalties: Elsevier (Editor, Braunwald's Heart Disease); site co-investigator: Abbott, Biotronik, Boston Scientific, CSI, Endotronix, St. Jude Medical (now Abbott), Philips, SpectraWAVE, Svelte, Vascular Solutions; trustee: American College of Cardiology; unfunded research: FlowCo, Takeda. V.A.B. reports grant support from Sanofi, Regeneron Pharmaceuticals, Amgen, Astra Zeneca, DalCor, Esperion, and Novartis; consulting fees from Pfizer; honoraria from Medscape; and fees for participating on Data Safety Monitoring Boards for the NIH and for Verve Therapeutics. R.D. reports research grants from Sanofi, DalCor Pharmaceuticals, Population Health Research Institute, Duke Clinical Research Institute, the TIMI group, Amgen, Cirius, Montreal Health Innovations Coordinating Center, and Lepetit; and personal fees, as a member of the Executive Steering Committee, from Amgen and Cirius. R.A.H. reports research grants from the Patient-Centered Outcomes Research Institute, NIH, CSL and Janssen; consulting for Atropos Health, Bitterroot Bio, Bridge Bio, Bristol Myers Squibb, Foresight, Element science; and serving on the boards of directors for the American Heart Association (unpaid) and Cytokinetics. J.W.W. reports research grants from the Netherlands Heart Foundation, the Interuniversity Cardiology Institute of the Netherlands, and the European Commission Seventh Framework Programme; and research support from Amgen, Astellas, AstraZeneca, Daiichi-Sankyo, Lilly, Merck–Schering-Plough, Pfizer, Roche, and Sanofi. H.D.W. reports grant support paid to the institution for serving on a steering committee for the ODYSSEY OUTCOMES trial (Evaluation of Cardiovascular Outcomes After an Acute Coronary Syndrome During Treatment With Alirocumab) from sanofi-aventis and Regeneron Pharmaceuticals, for the ACCELERATE study (A Study of Evacetrapib in High-Risk Vascular Disease) from Eli Lilly and Company, for the STRENGTH trial (Outcomes Study to Assess Statin Residual Risk Reduction With EpaNova in High CV Risk Patients With Hypertriglyceridemia) from Omthera Pharmaceuticals, for the CAMELLIA-TIMI study [A Study to Evaluate the Effect of Long-term Treatment With BELVIQ (Lorcaserin HC) on the Incidence of Major Adverse Cardiovascular Events and Conversion to Type 2 Diabetes Mellitus in Obese and Overweight Subjects With Cardiovascular Disease or Multiple Cardiovascular Risk Factors] from Eisai Inc., for the HEART-FID study (Randomized Placebo-Controlled Trial of FCM as Treatment for Heart Failure With Iron Deficiency) from American Regent, and for the ISCHEMIA Trial (International Study of Comparative Health Effectiveness With Medical and Invasive Approaches) and the MINT Trial (Myocardial Ischemia and Transfusion) from the NIH USA. He also received grants to the institution and personal fees as a steering committee member for the dal-GenE study (Effect of Dalcetrapib vs. Placebo on CV Risk in a Genetically Defined Population With a Recent ACS) from DalCor Pharma UK Inc., for the AEGIS-II study (The Safety and Tolerability of CSL112, a Reconstituted, Infusible, Plasma-Derived Human ApoA-I, After Acute Myocardial Infarction: The ApoA-I Event reducinG in Ischemic Syndromes I) from CSL Behring, for the SCORED trial (Effect of Sotagliflozin on Cardiovascular and Renal Events in Patients With Type 2 Diabetes and Moderate Renal Impairment Who Are at Cardiovascular Risk) and the SOLOIST-WHF trial (Effect of Sotagliflozin on Cardiovascular Events in Patients With Type 2 Diabetes Post Worsening Heart Failure) from sanofi-aventis Australia Pty Ltd, and for the CLEAR Outcomes Study [Evaluation of Major Cardiovascular Events in Patients With, or at High Risk for, Cardiovascular Disease Who Are Statin Intolerant Treated With Bempedoic Acid (ETC-1002) or Placebo] from Esperion Therapeutics Inc. H.D.W. was on the Advisory Boards for CSL Behring and Genentech, Inc. (an affiliate of F. Hoffmann-La Roche Ltd, ‘Roche’; Lytics Post-PCI Advisory Board at European Society of Cardiology. A.M.Z. reports receiving fees for serving on a steering committee for the ODYSSEY OUTCOMES trial from Sanofi, and advisory board and speaker fees from Sanofi, Amgen, Boehringer Ingelheim, Bayer, Novartis, Pfizer, AstraZeneca, and Vifor. G.M. and R.P. are employees of Regeneron Pharmaceuticals, Inc. Y.P. is an IT&M Stats employee and IT&M Stats reports consultancy fees from Sanofi. W.S. is an employee of Sanofi and may hold shares and/or stock options in the company. G.G. is an employee of Sanofi and may hold shares and/or stock options in the company. G.G.S. reports research support (all paid to institution) from Sanofi; grants or contracts from AstraZeneca, Silence Therapeutics, and The Medicines Company (all paid to institution); support for attending meetings from University of Oxford, American Society of Preventive Cardiology, and Cardiovascular Clinical Trialists; and receipt of medical writing support from Novartis.

## Data availability

The dataset(s) supporting the conclusions of this article is(are) included within the article.

## Notes

The ODYSSEY OUTCOMES committee members, investigators, and contributors are listed in Supplementary material online.
